# The lactose operon from *Lactobacillus casei* is involved in the transport and metabolism of the human milk oligosaccharide core-2 *N*-acetyllactosamine

**DOI:** 10.1038/s41598-018-25660-w

**Published:** 2018-05-08

**Authors:** Gonzalo N. Bidart, Jesús Rodríguez-Díaz, Gaspar Pérez-Martínez, María J. Yebra

**Affiliations:** 1 Laboratorio de Bacterias Lácticas y Probióticos, Departamento de Biotecnología de Alimentos, IATA-CSIC, Valencia, Spain; 2 Departamento de Microbiología, Facultad de Medicina, Universidad de Valencia, Valencia, Spain

## Abstract

The lactose operon (*lacTEGF*) from *Lactobacillus casei* strain BL23 has been previously studied. The *lacT* gene codes for a transcriptional antiterminator, *lacE* and *lacF* for the lactose-specific phosphoenolpyruvate: phosphotransferase system (PTS^Lac^) EIICB and EIIA domains, respectively, and *lacG* for the phospho-β-galactosidase. In this work, we have shown that *L. casei* is able to metabolize *N*-acetyllactosamine (LacNAc), a disaccharide present at human milk and intestinal mucosa. The mutant strains BL153 (*lacE*) and BL155 (*lacF*) were defective in LacNAc utilization, indicating that the EIICB and EIIA of the PTS^Lac^ are involved in the uptake of LacNAc in addition to lactose. Inactivation of *lacG* abolishes the growth of *L. casei* in both disaccharides and analysis of LacG activity showed a high selectivity toward phosphorylated compounds, suggesting that LacG is necessary for the hydrolysis of the intracellular phosphorylated lactose and LacNAc. *L. casei* (*lacAB*) strain deficient in galactose-6P isomerase showed a growth rate in lactose (0.0293 ± 0.0014 h^−1^) and in LacNAc (0.0307 ± 0.0009 h^−1^) significantly lower than the wild-type (0.1010 ± 0.0006 h^−1^ and 0.0522 ± 0.0005 h^−1^, respectively), indicating that their galactose moiety is catabolized through the tagatose-6P pathway. Transcriptional analysis showed induction levels of the *lac* genes ranged from 130 to 320–fold in LacNAc and from 100 to 200–fold in lactose, compared to cells growing in glucose.

## Introduction

All human milk oligosaccharides (HMOs) contain lactose (Galβ1-4Glc) at the reducing end, which can be elongated with lacto-*N*-biose (LNB, type-1 core; Galβ1-3GlcNAc) by a β-1,3 bond and/or with *N*-acetyllactosamine (LacNAc, type-2 core; Galβ1-4GlcNAc) by a β-1,3 or β-1,6-bond^1,2^. Recently, LacNAc has also been found as a free disaccharide in human milk, with concentrations that decrease from 310 μg/ml on colostrum to 6.7 μg/m after the first week of lactation^3^. LacNAc is also a common structure of human glycans present at mucosal surfaces and other specific tissues and cells^4^. The LacNAc residues usually constitute the terminal sugars that form part of the variable portions of the glycan epitopes, including *O*-glycans, *N*-glycans and glycolipids. Sometimes these proteins and lipids carry poly-*N*-acetyllactosamines chains, which can be acceptors for subsequent glycosylations and serve as specific arms to present other functional terminal glycans^4^. Additionally, LacNAc is a constituent of the ABO and Lewis blood group antigens, which are expressed on the membrane of blood red cells and other tissues, including the gastrointestinal epithelium^5^.

**Figure 1:**
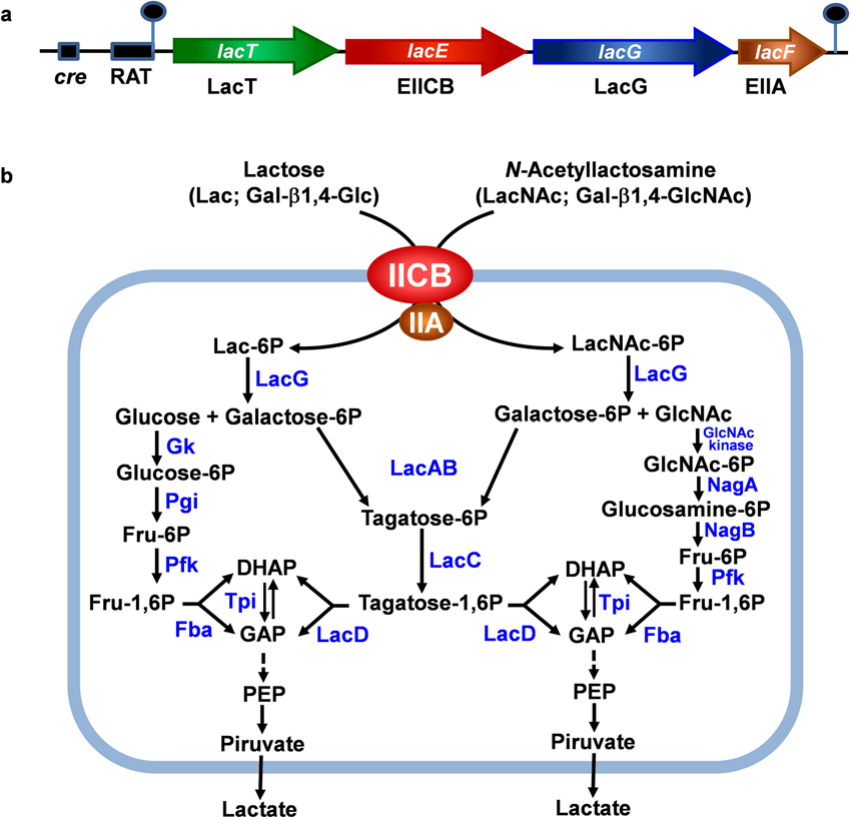
(**a**) Genetic organization of the *lac* operon in *Lactobacillus casei* strain BL23. Hairpin loops indicate *rho*-independent transcriptional terminators. *cre*, catabolite responsive element; RAT, ribonucleic antiterminator; LacT is a transcriptional antiterminator; EIICB and EIIA, domains of the lactose-specific phosphoenolpyruvate: phosphotransferase system (PTS^Lac^); LacG, phospho-β-galactosidase. (**b**) Schematic presentation of the pathways for *N*-acetyllactosamine (LacNAc) and lactose (Lac) transport and metabolism in *L. casei* BL23. GlcNAc: *N*-acetylglucosamine; Fru, fructose; DHAP: dyhidroxyacetone phosphate; GAP: glyceraldehyde 3-phosphate; PEP: phosphoenolpyruvate; NagA, *N*-acetylglucosamine-6P deacetylase; NagB: glucosamine-6P deaminase; Pfk: 6-phosphofructo-1-kinase; Gk, glucokinase; Pgi, phosphoglucose isomerase; LacAB: galactose-6P isomerase; LacC: tagatose-6P kinase; LacD: tagatose-1,6 P aldolase; Fba: fructose-1,6 P aldolase; Tpi: triose phosphate isomerase.

The gastrointestinal tract of breast-fed infants is rapidly colonized by *Bifidobacterium* species^6^, which are well adapted to metabolize HMOs^7^. This capacity has been associated with a complete array of enzymes that includes various types of intra- and extracellular glycosidases^8–10^. LacNAc and other LacNAc-containing oligosaccharides, such as lacto-*N*-neotetraose and lacto-*N*-hexaose, are digested *in vitro* by the extracellular membrane-bound β-galactosidase BbgIII cloned from *Bifidobacterium bifidum*^11,12^. This species also contains three intracellular β-glycosidases (BbgI, BbgII and BbgIV) able to hydrolyze LacNAc *in vitro*, although different from BgbIII, they have higher affinity for lactose than for LacNAc^11,12^. Galactose is also liberated from the LacNAc contained in the lacto-*N*-neotetraose structure by β-galactosidases described in *Bifidobacterium longum* and *Bifidobacterium breve* strains^13^. The genus *Lactobacillus* has many characterized probiotic strains^14,15^ and some species have also been isolated from the gastrointestinal tract of infants^16,17^. However, contrarily to *Bifidobacterium* species, genome analysis of lactobacilli revealed that they have a limited capability to ferment HMOs or mucosa-derived glycans^18–20^. One exception to this are the members of the *Lactobacillus casei/paracasei/rhamnosus* phylogenetically close group. We have previously characterized in *L. casei* three genes encoding α-L-fucosidases and purified the corresponding enzymes, which hydrolyze *in vitro* fucosylated HMOs^21^. Specifically, the operon *alf*, encoding the α-L-fucosidase AlfB, the transcriptional repressor AlfR and the EII components of a mannose-class phosphoenolpyruvate: phosphotransferase system (PTS), is involved in these species in the metabolism of fucosyl-α1,3-GlcNAc disaccharide, an structure present in HMOs and mucins^22,23^. The type-1 HMOs core LNB and the type-1 core *O*-glycosylation galacto-*N*-biose (GNB) are also metabolized by *L. casei*^24^. Both disaccharides are transported and phosphorylated by the PTS^Gnb^ and then are hydrolyzed by the phospho-β-galactosidase GnbG, which are encoded by the *gnb* operon^24^. Recently, we have characterized the cell-wall anchored *N*-acetylglucosaminidase BnaG necessary for the utilization of the trisaccharide lacto-*N*-triose by *L. casei*^25^. BnaG is the only extracellular glycosidase described until now for the metabolism of HMOs in lactobacilli. *L. casei* also metabolizes lactose and the *lac* operon (Fig. 1a) from *L. casei* strain BL23 has been extensively studied in our laboratory^26–29^. The *lacT* gene codes for a transcriptional antiterminator, *lacE* and *lacF* genes for the lactose-specific PTS, EIICB and EIIA domains, respectively, and *lacG* for the phospho-β-galactosidase^27^. The *lac* operon is induced by lactose through transcription antitermination mediated by LacT^28^. Additionally, the expression of this operon is subject to carbon catabolite repression mediated by the general regulatory protein CcpA (catabolite control protein A) and also by PTS elements via LacT^26,28^. Despite the relevance of the LacNAc disaccharide, its metabolism has not been studied in the genus *Lactobacillus*. Here we report that *L. casei* strain BL23 is able to grow in the presence of LacNAc and that the *lac* operon is involved in its metabolism. Additionally, we demonstrated that the tagatose-6P pathway and the *N*-acetylglucosamine-6P deacetylase NagA are involved in the catabolism of this disaccharide.

## Results

### *L. casei* BL23 can be cultured in the presence of LacNAc and transports it by the PTS^Lac^

**Figure 2:**
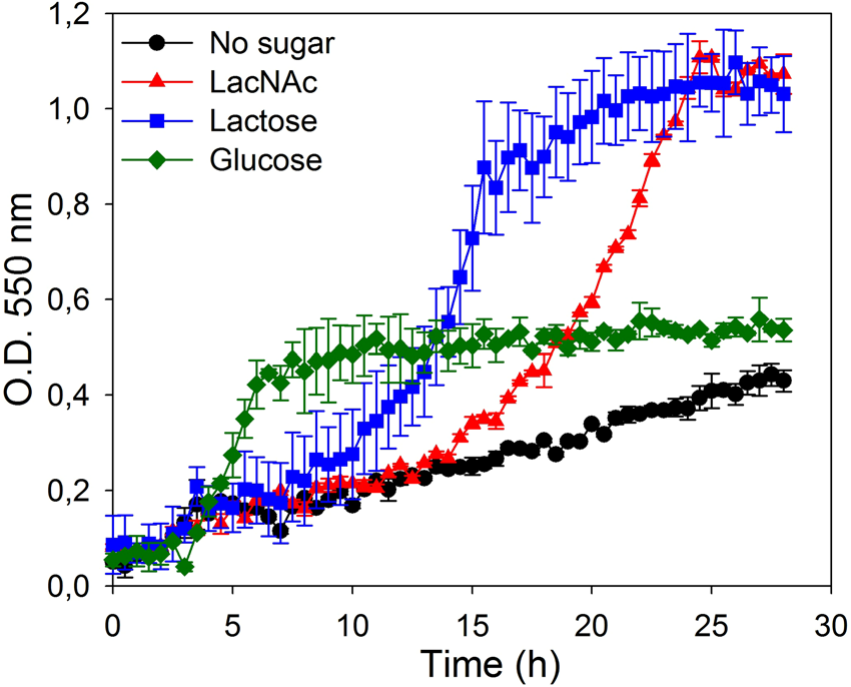
Growth curves of *Lactobacillus casei* wild type strain BL23 on sugar-free MRS without carbon source (black), with *N*-acetyllactosamine (LacNAc) (red), lactose (blue) or glucose (green). Data presented are mean values based on at least three replicates. Error bars indicate standard deviations.

We have previously shown that *L. casei* is able to transport and ferment LNB, the type-1 disaccharide building block of HMOs^24^. In order to determine the ability of this species to metabolize the type-2 core structure of HMOs, we cultured *L. casei* BL23 in sugar-free MRS supplemented with 4 mM LacNAc as carbon source (Fig. 2). The results showed that *L. casei* can grow in the presence of this disaccharide and that the maximum cell density reached is similar to that obtained in lactose, which was used as a positive control.

**Figure 3:**
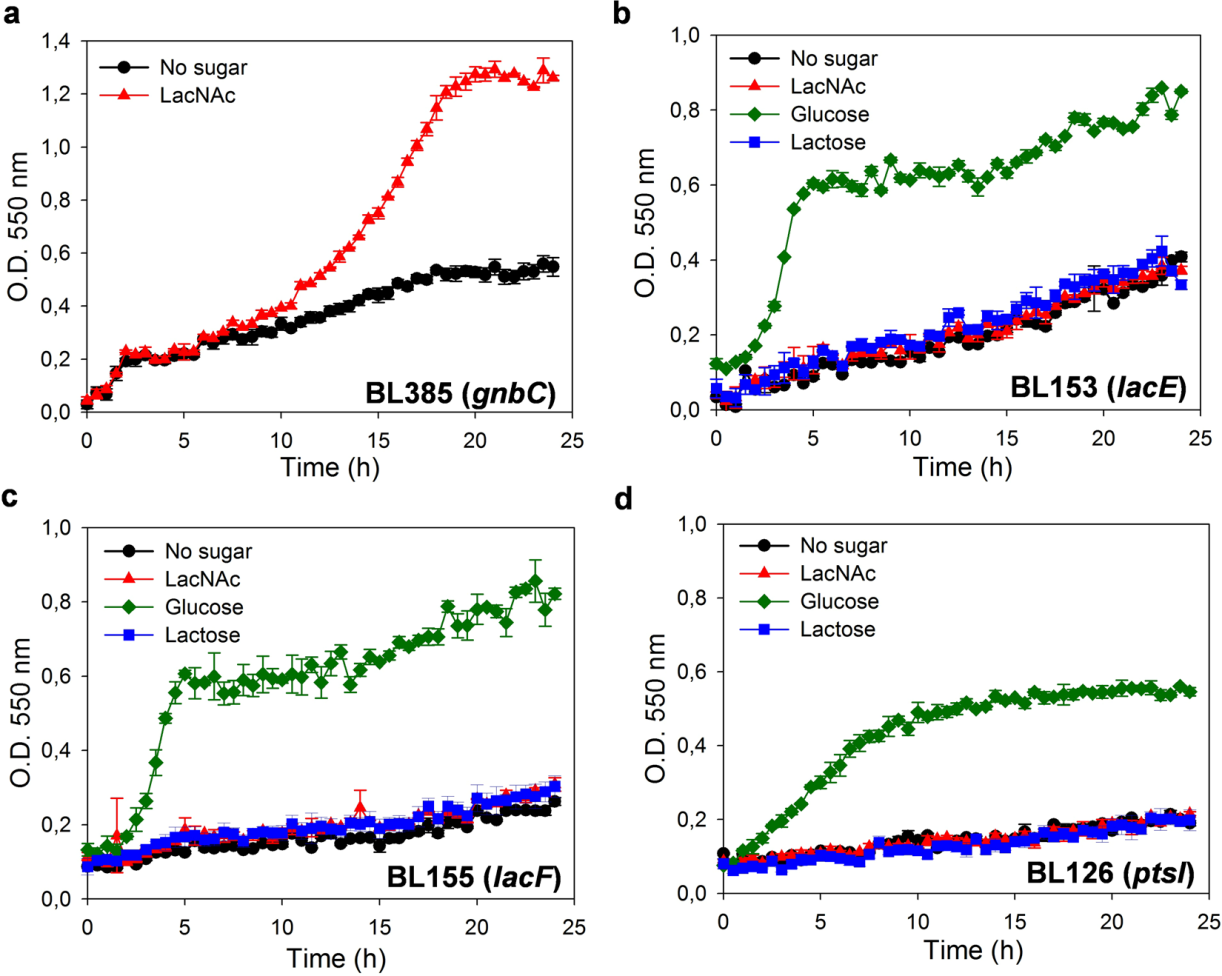
Growth curves of *Lactobacillus casei* mutant strain BL385 (*gnbG*) (**a**) on sugar-free MRS without carbon source (black) or with *N*-acetyllactosamine (LacNAc) (red). Data presented are mean values based on at least three replicates. Error bars indicate standard deviations. Growth curves of *L. casei* mutant strain BL153 (*lacE*) (**b**), *L. casei* mutant strain BL155 (*lacF*) (**c**) and *L. casei* mutant strain BL126 (*pstI*) (**d**) on sugar-free MRS without carbon source (black), with *N*-acetyllactosamine (LacNAc) (red), glucose (green) or lactose (blue). Data presented are mean values based on at least three replicates. Error bars indicate standard deviations.

Most of the characterized PTS are specific for one carbohydrate, however some PTS can transport two or more structurally related sugars^30–32^. Therefore, we analyzed if LacNAc, which is structurally similar to LNB and lactose, is transported in *L. casei* BL23 by the PTS^Gnb^ or by the PTS^Lac^, which are involved in LNB and lactose uptake, respectively^24,28^. The mutant strains BL385 (*gnbC*)^24^, that is disrupted in the gene encoding the EIIC domain of the PTS^Gnb^, BL153 (*lacE*) and BL155 (l*acF*)^28^, which are impaired in the EIICB and EIIA, respectively, of the PTS^Lac^, were tested for their capacity to ferment LacNAc. BL385 (*gnbC*) was able to grow in the presence of LacNAc as carbon source (Fig. 3a). Contrarily, strains BL153 (*lacE*) and BL155 (*lacF*) showed a poor growth with LacNAc which was similar to that of the negative controls (lactose supplemented and non-supplemented sugar-free MRS) (Fig. 3b,c). The growth pattern of strains BL153 (*lacE*) and BL155 (*lacF*) in the presence of glucose as a positive control is also shown (Fig. 3b,c). Analysis for sugar content in the supernatants demonstrated that LacNAc was consumed by strain BL385 (*gnbC*) but not by strains BL153 (*lacE*) and BL155 (*lacF*) (data not shown), indicating that the domains EIICB and EIIA encoded by *lacE* and *lacF*, respectively, are involved in the uptake of LacNAc (Fig. 1). It has been previously shown that the PTS from *L. casei* can accomplish sugar transport not coupled to phosphorylation^23^. Then, to further confirm the involvement of the PTS^Lac^ in the transport of LacNAc and to test if transport though the EII permease was coupled to phosphorylation, the growth pattern of BL126 (*ptsI*), a mutant lacking the PTS-general component Enzyme I^33^, was tested in LacNAc as carbon source. BL126 (*ptsI*) did not grow in the presence of LacNAc (Fig. 3d), confirming that its utilization needs a functional complete PTS. These results suggest that LacNAc is internalized as a phosphorylated derivative. BL126 (*ptsI*) was grown with glucose and lactose as positive and negative controls, respectively. The growth pattern of strain BL126 in the presence of glucose differs from that of strains BL153 (*lacE*) and BL155 (*lacF*) (Fig. 3). This might be due to the fact that the latest strains contain a functional PTS for glucose uptake while strain BL126 (*ptsI*) can only transport this sugar by the proton-driven permease^34^.

### LacG is involved in the metabolism of LacNAc and lactose

**Figure 4:**
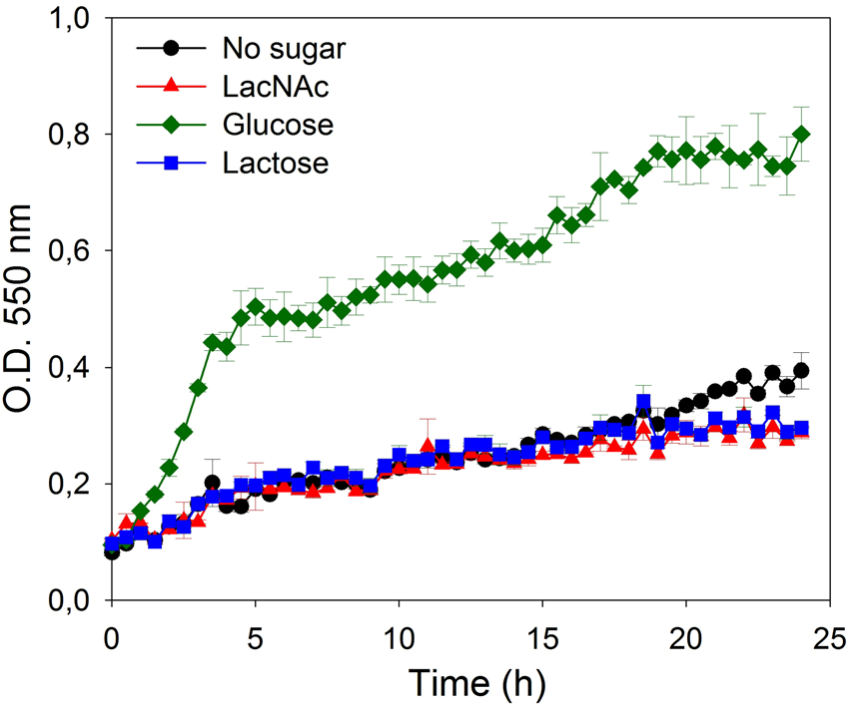
Growth curves of *Lactobacillus casei* mutant strain BL400 (*lacG*) on sugar-free MRS without carbon source (black), with *N*-acetyllactosamine (LacNAc) (red), glucose (green) or lactose (blue). Data presented are mean values based on at least three replicates. Error bars indicate standard deviations.

To determine if the phospho-β-galactosidase LacG was involved in the utilization of LacNAc in *L. casei* BL23, a mutant in *lacG* was constructed (strain BL400). This mutant was cultured in sugar-free MRS supplemented with 4 mM LacNAc as carbon source (Fig. 4). The growth pattern showed that BL400 (*lacG*) strain did not ferment LacNAc and neither did lactose, the only substrate described until now for the *lac* operon^26–28^. These results indicated that LacG is necessary for the utilization of both disaccharides (Fig. 1b). Sugar content analysis of the culture supernatants detected LacNAc and lactose, respectively, in the supernatants from BL400 (*lacG*), while they were completely consumed by the wild-type BL23 strain (data not shown).

LacG (EC 3.2.1.85) belongs to the glycosyl hydrolase family 1 (GH 1; www.cazy.org), which includes β-glycosidases as well as phospho-β-glycosidases. In order to characterize that enzyme, the *lacG* gene was expressed in *E. coli* as a His-tagged protein and purified to homogeneity (data not shown). The purified protein displayed a molecular weight of 55 kDa, in agreement with the calculated mass of the 6x(His)-tagged protein (55,305 Da). 6x(His)LacG did not hydrolyze *o*-NP-β-D-galactopyranoside but it does when this substrate is phosphorylated (Table 1), suggesting a high selectivity toward phosphorylated compounds. The kinetic analysis showed a high *Km* and low *Vmax* for *o*-NP-β-D-galactopyranoside-6P, and it displayed an optimal pH of 7.0 and an optimal temperature of 41 °C (Table 1). 6x(His)LacG was unable to hydrolyze any of the natural oligosaccharides tested (Table 1), including lactose and LacNAc, possibly because these need to be phosphorylated before turned into substrates for this glycosidase.

**Table 1:** Activity and characterization of the phospho-β-galactosidase LacG.

Substrate^a^ (Structure)	Activity^b^
*o*-NP-β-D-galactopyranoside	−
*p*-NP-β-D-glucopyranoside	−
*o*-NP-1-thio-β-D-galactopyranoside	−
*p*-NP-β-D-glucuronide	−
*p*-NP-*N*-acetyl-β-D-glucosaminide	−
*p*-NP-α-D-glucopyranoside	−
*p*-NP-α-D- galactopyranoside	−
*p*-NP-α-L- fucopyranoside	−
*o*-NP-β-D-galactopyranoside-6-phosphate	+
Lacto-*N*-biose (Galβ1-3GlcNAc)	−
Galacto-*N*-biose (Galβ1-3 GalNAc)	−
Lacto-*N*-tetraose (Galβ1-3GlcNAcβ1-3 Galβ1-4Glc)	−
Lacto-*N*-neotetraosa (Galβ1-4GlcNAcβ1-3 Galβ1-4Glc)	−
3′-*N*-Acetilgalactosaminil-Gal (GalNAcβ1-3Gal)	−
3′-*N*-Acetilglucosaminil-Man (GlcNAcβ1-3Man)	−
4′-Galactofuranosil-GlcNAc (Galβ1-4GlcNAc)	−
Fucosyl-α1-3GlcNAc	−
Lactose (Galβ1-4Glc)	−
*N*-acetyl-lactosamine (Galβ1-4GlcNAc)	−
Lactulose (Galβ1-4Fru)	−
Maltose (Glcα1-4Glc)	−
Maltotriose (Glcα1-4Glcα1-4Glc)	−
Characterization^c^	
*V*_*max*_ (μmol (mg protein^−1^ min^−1^)	1.4
*K*_*m*_ (mM)	3.0
Optimal pH	7.0
Optimal temperature (°C)	41

^a^Carbohydrates used as substrates. NP, nitrophenyl; Glc, glucose; Gal, Galactose; GlcNAc, *N*-acetylglucosamine; GalNAc, *N*-acetylgalactosamine; Man, mannose; Fru, fructose.

^b^+, substrate is totally hydrolyzed after 16 h reaction in the conditions described in the “Materials and methods” section; −, no activity detected.

^c^The enzyme activity was determined with *o*-nitrophenyl-β-D-galactopyranoside-6-P as the substrate.

### The tagatose-6P pathway and the *N*-acetylglucosamine-6P deacetylase NagA are involved in the metabolism of LacNAc

**Figure 5:**
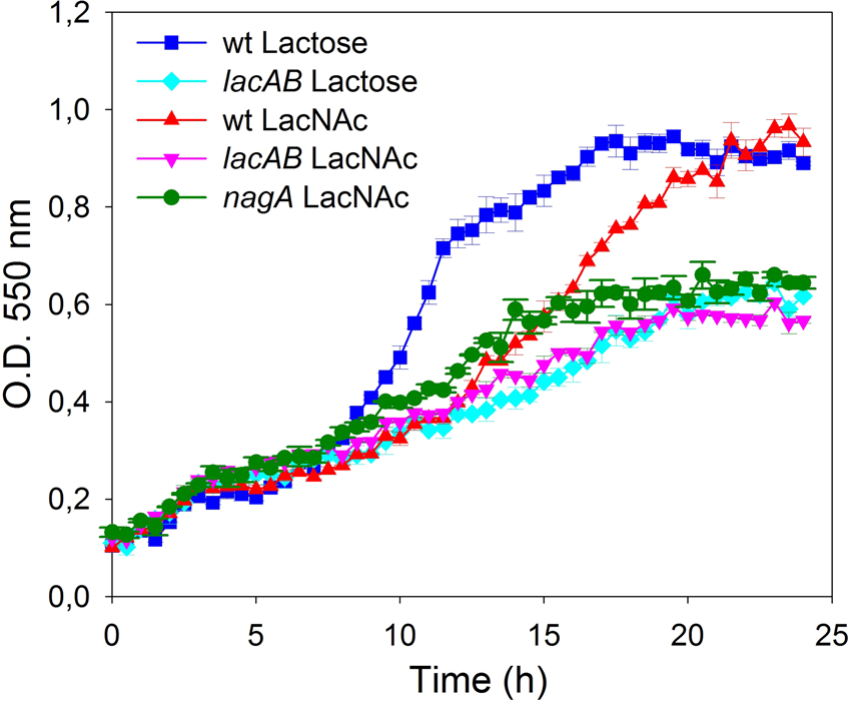
Growth curves of *Lactobacillus casei* wild type strain BL23 (blue) and mutant strain BL393 (*lacAB*) (cyan) on sugar-free MRS with lactose. BL23 (red) and mutants BL393 (*lacAB*) (pink) and BL388 (*nagA*) (green) on sugar-free MRS supplemented with *N*-acetyllactosamine (LacNAc). Data presented are mean values based on at least three replicates. Error bars indicate standard deviations.

The results described above suggest that, as occurs with lactose, LacNAc is transported and phosphorylated by the PTS^Lac^, and then hydrolyzed by the phospho-β-galactosidase LacG into GlcNAc and Gal-6P. It has been assumed that this phosphorylated sugar resulting from the lactose metabolism is catabolized through the Tag-6P pathway in *L. casei*^35,36^. To analyze this at the genetic level, the mutant strain *L. casei* BL393 (*lacAB*), deficient in the heteromeric Gal-6P isomerase of the Tag-6P route^24^, was cultured on lactose or LacNAc (Fig. 5). This strain showed a growth rate in lactose (0.0293 ± 0.0014 h^−1^) and LacNAc (0.0307 ± 0.0009 h^−1^) significantly lower than the wild-type on these disaccharides (0.1010 ± 0.0006 h^−1^ and 0.0522 ± 0.0005 h^−1^, respectively) (wild-type versus *lacAB* mutant, *P* = 0.0004 (lactose); *P* = 0.0012 (LacNAc)). These results supported that lactose and LacNAc metabolism in *L. casei* utilizes the Tag-6P pathway for the catabolism of the galactose moiety (Fig. 1b). Additionally, these results suggest that the residual growth showed by BL393 (*lacAB*) strain on those carbohydrates would be maintain by the catabolism of the glucose and GlcNAc moieties, resulting from the lactose and LacNAc hydrolysis, respectively.

We have previously shown that in *L. casei* the *nagA* gene, encoding an *N*-acetylglucosamine-6P deacetylase, is involved in the metabolism of GlcNAc either free o derived from LNB^24^. Here, we tested the growth of the mutant strain *L. casei* BL388 disrupted in *nagA* on LacNAc as carbon source (Fig. 5). The results showed a growth rate for this mutant (0.0436 ± 0.0008 h^−1^) significantly lower (*P* = 0.004) than the wild-type (0.0522 ± 0.0005 h^−1^), suggesting that *nagA* is required for the catabolism of the GlcNAc moiety resulting from the hydrolysis of this disaccharide.

### Transcriptional analyses of the *lac* operon

**Figure 6:**
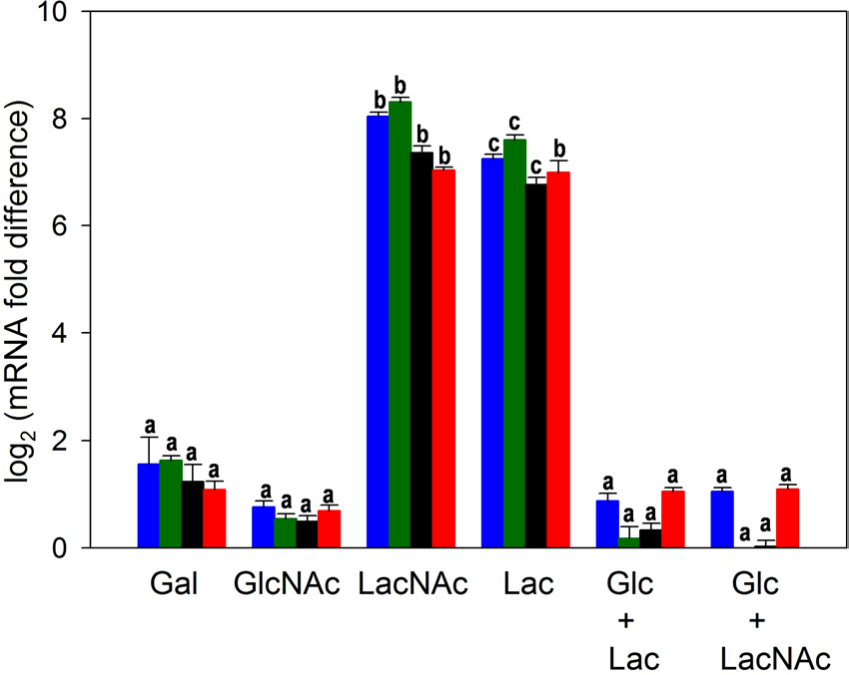
Transcriptional analysis by RT-qPCR of the expression of *lacT* (blue bars), *lacE* (green bars), *lacG* (black bars) and *lacF* (red bars) in *Lactobacillus casei* wild type strain BL23 grown in sugar-free MRS containing galactose (Gal), *N*-acetylglucosamine (GlcNAc), *N*-acetyllactosamine (LacNAc), lactose (Lac), a mix of glucose and lactose (Glc + Lac) or a mix of glucose and *N*-acetyllactosamine (Glc + LacNAc). Cells grown in sugar-free MRS supplemented with glucose were used as reference condition. Data presented are mean values based on three replicates of at least two biological independent samples. Bars indicate standard errors. For each *lac* gene, significantly different values (*P* < 0.05) among culture conditions are marked by different lower-case letters.

Northern blot analyses have previously shown that the *lacTEGF* operon from *L. casei* is induced by lactose and subjected to carbon catabolite repression by glucose^28^. In order to determine if the transcription of the *lac* genes are also regulated by LacNAc, RNA was isolated from *L. casei* wild-type strain BL23 grown in sugar-free MRS containing galactose, GlcNAc, glucose, LacNAc or LacNAc plus glucose and used for RT-qPCR analyses. Additionally, RNA obtained from cultures grown on lactose or lactose plus glucose were also included in these analyses to quantify the expression levels of the *lac* genes in these carbon sources (Fig. 6). Taking as a reference the transcript levels in cells growing in glucose, the *lacT, lacE, lacG* and *lacF* were induced by LacNAc and lactose. The induction levels ranged from 130 to 320–fold and from 100 to 200–fold in LacNAc and lactose, respectively. The expression levels were highly reduced when these carbohydrates were mixed with glucose (Fig. 6). These results indicate that the *lac* operon in addition to lactose is also induced by LacNAc and confirmed that it is repressed by glucose. The *lac* genes were barely expressed in the presence of galactose and GlcNAc, denoting that their induction relies on the presence of the disaccharide and not on the monosaccharides resulting from the hydrolysis.

### The transcriptional antiterminator LacT is required for LacNAc metabolism

**Figure 7:**
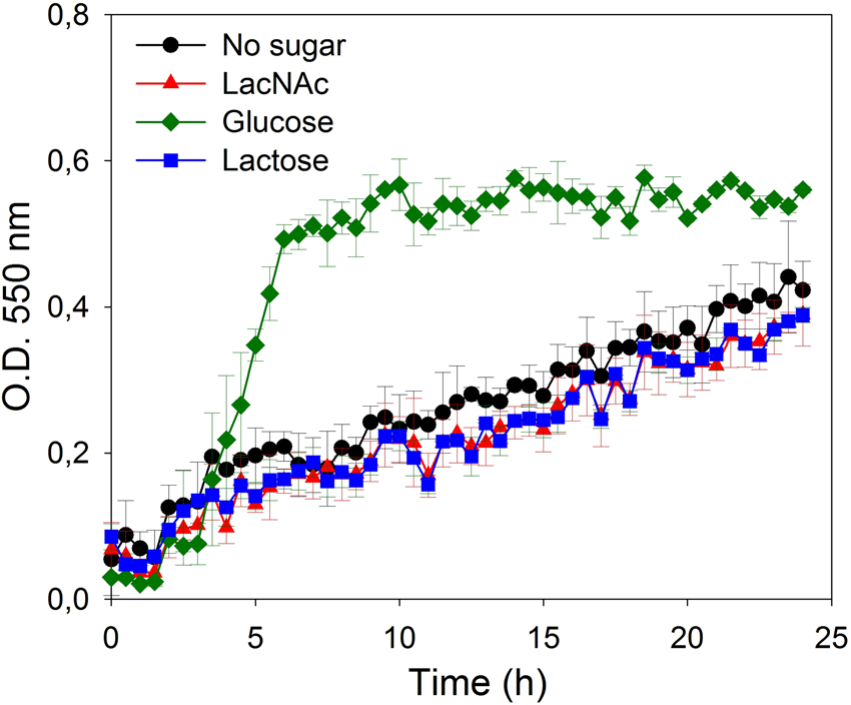
Growth curves of *Lactobacillus casei* mutant strain BL195 (*lacT*) on sugar-free MRS without carbon source (black), with *N*-acetyllactosamine (LacNAc) (red), glucose (green) or lactose (blue). Data presented are mean values based on at least three replicates. Error bars indicate standard deviations.

The *L. casei* LacT protein prevents transcription termination of the *lac* operon in response to the presence of lactose in the culture medium^26^. In order to determine the involvement of this transcriptional antiterminator in the metabolism of LacNAc, the mutant strain *L. casei* BL195 (*lacT*), deficient in LacT^26^, was cultured on LacNAc as carbon source (Fig. 7). BL195 (*lacT*) strain was also grown with glucose and lactose as positive and negative controls, respectively. The results show that this mutant strain exhibited a growth in the presence of LacNAc similar to that of the negative controls (lactose supplemented and non-supplemented sugar-free MRS), indicating that the transcriptional antitermiator LacT is also involved on LacNAc metabolism. Additionally, the results suggest that LacT antiterminates transcription of *lac* operon not only depending on the presence of lactose if not also on the presence of LacNAc in the growth medium.

## Discussion

The disaccharide *N*-acetyllactosamine (LacNac) has an important role in many cell recognition processes such as parasite-host cell interaction^37^, autoimmune^38^ and inflammatory^39^ diseases, and also in cancer^40^. Additionally, LacNAc is a key structure present at human milk oligosaccharides and also at the glycan domains of glycoproteins and glycolipids present in the gastrointestinal tract^41–43^. We have demonstrated that *L. casei* is able to metabolize LacNAc. Curiously, the *lac* operon, which has been widely studied in this strain^26–28^, is also the responsible of the transport and catabolism of LacNAc. As previously described for the PTS^Gnb^, that is involved in the transport of *N*-acetyl-galactosamine, LNB and GNB in *L. casei*^24^, the PTS^Lac^ represents a new example of a PTS able to transport two structurally related substrates, lactose and LacNAc. *L. casei* mutants deficient in either EIICB^Lac^ or EIIA^Lac^ ^28^, were unable to grow in the presence of LacNAc. Due to the great biotechnological and economic importance of lactose fermentation in the dairy industry there are a great number of studies directed to analyze lactose fermentation by lactic acid bacteria^44–47^. Lactose in these bacteria can be transported through proton symport permeases, lactose/galactose antiport systems^44,48^ or via PTS^28,49–51^. Analysis of the genome sequence of lactobacilli (http://www.ncbi.nlm.nih.gov/genomes) showed that genes encoding PTSs homologous to the PTS^Lac^ from *L. casei* BL23, are present in the *Lactobacillus casei/paracasei/rhamnosus/zeae* group of phylogenetically related lactobacilli, and also in a few strains of *Lactobacillus heilongjiangensis*, *Lactobacillus futsaii*, *Lactobacillus farciminis*, *Lactobacillus perolens*, *Lactobacillus fermentum*, *Lactobacillus sharpeae*, *Lactobacillus crustorum, Lactobacillus pobuzihii, Lactobacillus kimchiensis, Lactobacillus ruminis, Lactobacillus gasseri* and *Lactobacillus johnsonii*, suggesting that lactose-specific PTSs are widely distributed among lactobacilli. For *L. gasseri* strain ATCC 33323, which is autochthonous of the gut, it has already been demonstrated that it contains two different PTSs involved in the transport of lactose and that the expression of both was induced by this carbohydrate^51^. Another strain indigenous of the gut is *L. rhamnosous* TCELL-1, for which it has also been demonstrated that the *lac* operon is induced by lactose^50^. Whether those PTS are functional for lactose and LacNAc remains to be investigated. Transcriptional analysis demonstrated here that the *lac* operon from *L. casei* is induced by lactose as well as by LacNAc and that induction levels are higher on LacNAc than on lactose. This might suggest that LacNAc is the substrate to which the *lac* operon had been adapted first. Indeed, many *L. casei* strains have been isolated from the human gastrointestinal tract^52,53^, which is very rich in LacNAc^5,41^. In *E. coli*, the *lac* operon is not regulated directly by lactose if not by allolactose, a transient product synthesized by the β-galactosidase, and this has generated controversy about the true physiological role of the *lac* operon in this bacterium^54^.

*L. casei* transport LacNAc or lactose through the PTS^Lac^ resulting in the formation of LacNAc-P or lactose-P, which are further hydrolyzed inside the cell by the phospho-β-galactosidase LacG into Gal-6P and GlcNAc or glucose, respectively (Fig. 1b). We have biochemically characterized this enzyme and confirmed that can only hydrolyze phosphorylated substrates. This also occurs with all the proteins homologs to LacG that have been characterized until now, which have been isolated from *Streptococcus mutants*^55^, *Saphylococcus aureus*, *Lactococcus lactis*, *L. casei* strain 64H^56^ and *L. gasseri*^57^. Contrarily, β-galactosidases isolated from *Bifidobacterium bifidum* are able to hydrolyze non-phosphorylated lactose and LacNAc^11,12^, showing two different mechanisms to metabolize these disaccharides in species that would compete for them in environmental niches such as the gastrointestinal tract. The Gal-6P generated after the hydrolysis of LacNAc-P or lactose-P by LacG is channeled through the Tag-6P pathway. We have showed that the mutant strain *L. casei* BL393 (*lacAB*) is impaired in the growth on lactose and LacNAc. The genes encoding the Tag-6P route in *L. casei* are present in the operon *lacR1ABD2C*, which includes a transcriptional regulator LacR1, the two subunits of the heteromeric Gal-6P isomerase (*lacAB*), a Tag-6P kinase (*lacC*) and a Tag-1,6-bisP aldolase (*lacD2*). Unlike *L. casei*, in *Lactococcus lactis*^58^ and *Streptococcus mutans*^59^, the *lac* operon contains the genes *lacRABCDFEG* encoding the Tag-6P catabolic proteins in addition to the lactose-specific PTS and the phospho-β-galactosidase LacG.

*L. casei* species have been isolated from dairy products, plant material and reproductive and gastrointestinal tracts of humans and animals^52,53^, which reveals their great adaptability to different environments. Genome analyses have showed that gene loss and acquisition are the main events resulting in niche adaptation^60^. Additionally, lactobacilli also contain genes involved in sugar uptake, metabolism and regulation grouped in genomic islands^61^. Lactose metabolism is well known in the dairy industry, but few data is found about its metabolism by the gastrointestinal microbiota. Lactose and LacNAc are constituents of HMO molecules that reach the breastfeeding infant gut microbiota, and LacNAc and poly-LacNAc molecules are also present in high amounts in the newborn gut^62,63^. Here we showed that *L. casei* metabolizes both disaccharides by using the same transport system and catabolic enzymes, which could be another niche adaptation mechanism of this bacterium to optimize the metabolic machinery minimizing energy consumption in a very competitive environment such as the gastrointestinal tract. Furthermore, the present work evidences that a catabolic pathway designed for survival of lactobacilli in the children gut has become an important tool for the development of dairy fermented products.

## Materials and Methods

### Strains, growth conditions and plasmids

The strains and plasmids used in this work are enumerated in Table 2. The *L. casei* strains were grown at 37 °C in MRS broth (Difco). *E. coli* was utilized as a cloning host and was grown in Luria-Bertani medium (Pronadisa) at 37 °C. *E. coli* DH10B transformants were selected with ampicillin (100 μg ml^−1^), *E. coli* BE50 with ampicillin (100 μg ml^−1^) and kanamycin (25 μg ml^−1^) and *L. casei* with erythromycin (5 μg ml^−1^). The vectors pRV300^64^ and pQE80 (Qiagen) were used for disruption of genes in *L. casei* and overproduction of proteins, respectively. *E. coli* strains were transformed by electroporation with a Gene Pulser apparatus (Bio-Rad Laboratories) as indicated by the manufacturer, and *L. casei* strains were transformed as described previously^65^.

**Table 2:** Strains and plasmids used in this study^a^.

Strain or plasmid	Relevant genotype or properties	Source or reference
**Strains**		
*Lactobacillus casei*		
BL23	Wild type	CECT 5275 (Acedo-Félix *et al*.^70^)
BL126	BL23 *ptsI*	Viana *et al*.^33^
BL153	BL23 *lacE*	Gosalbes *et al*.^28^
BL155	BL23 *lacF*	Gosalbes *et al*.^28^
BL195	BL23 *lacT*	Gosalbes *et al*.^26^
BL385	BL23 *gnbC*::pRV300 Erm^R^	Bidart *et al*.^24^
BL388	BL23 *nagA*::pRV300 Erm^R^	Bidart *et al*.^24^
BL393	BL23 *lacAB*	Bidart *et al*.^24^
BL400	BL23 *lacG* (frameshift at *Sph*I site)	This work
***Escherichia coli***		
DH10B	F^−^ *endA1 recA1 galE15 galK16 nupG rpsL* Δl*acX74* Φ80l*acZ*ΔM15 *araD139* Δ(*ara,leu*)7697 *mcrA* Δ(*mrr*) *sdRMS-mcrBC*) λ^−^	Invitrogen
BE50	BL21(DE3) containing pREPGroES/GroEL	Dale *et al*.^71^
PE172	BE50 containing pQElacG	This work
**Plasmids**		
pRV300	Suicide vector carrying Erm^R^ from pAMβ1	Leloup *et al*.^64^
pRVlacG	pRV300 with a frameshift at *Sph*I site in *lacG* fragment	This work
pQE80	*E. coli* expression vector; Amp^R^	Qiagen
pQElacG	pQE80 containing *lacG*-coding region	This work

^a^CECT, Colección Española de Cultivos Tipo; Erm^R^, erythromycin resistance; Amp^R^, ampicillin resistant.

### Culture of *L. casei* strains with lactose and LacNAc

The *L. casei* strains were cultured as previously described^24^ on sugar-free MRS containing: bactopeptone (Difco), 10 g l^−1^; yeast extract (Pronadisa), 4 g l^−1^; sodium acetate, 5 g l^−1^; tri-ammonium citrate, 2 g l^−1^; magnesium sulphate 7-hydrate, 0.2 g l^−1^; manganese sulphate monohydrate, 0.05 g l^−1^; and Tween 80, 1 ml l^−1^. LacNAc (Carbosynth, Compton, Berkshire, UK), lactose (Sigma-Aldrich, St. Louis, MO, USA), *N*-acetylglucosamine, galactose or glucose were added to the sugar-free MRS medium at a concentration of 4 mM. Bacterial growth was assayed in microtiter plates (100 μl culture broth per well) at 37 °C in a POLARstar Omega plate reader (BMG Labtech, Offenburg, Germany). The Gompertz model (GraphPad Software, San Diego, CA) was used for the analysis of the growth rates (μ).

### DNA manipulation and sequencing

DNA was obtained from *L. casei* BL23 as previously described^65^. Plasmid DNA was isolated from *E. coli* by using the kit illustra plasmidPrep Mini Spin (GE Healthcare, UK). Standard methods were used for recombinant DNA techniques^66^ and PCR reactions were carried out with the Expand High Fidelity PCR System (Roche). The DNA sequencing reactions were performed by the Central Service of Research Support of the University of Valencia (Spain). Specific primers hybridizing within the proper DNA fragments and universal primers were used for sequencing. The analysis of DNA sequences was performed with the aid of the DNAMAN 4.03 software package (Lynnon BioSoft) and sequence similarities were analyzed with the BLAST program^67^.

### Construction of a *lacG* mutant strain

A DNA fragment containing part of *lacG* was obtained by PCR using *L. casei* BL23 chromosomal DNA and the oligonucleotides lacGfor (5′-CAAGGAAGACGGTAAAGG) and lacGrev (5′-CCAACGGATAGTCATTATG). The PCR product was cloned into pRV300 digested with *Eco*RV. The resulting plasmid pRVlacG was cleaved at the unique *Sph*I restriction site, made blunt with the Klenow fragment, ligated and transformed to select a plasmid with a frameshift at the *Sph*I site in *lacG*. *L. casei* was transformed with this plasmid and one integrant where single recombination occurred was selected and cultured at 37 °C without antibiotic selection for at least 200 generations. Cells were grown on MRS-agar plates and screened for erythromycin-sensitive phenotype by replica plating on MRS-agar plates with erythromycin. Antibiotic-sensitive clones were selected and, among them, one was chosen (BL400 strain) in which a double-crossover event conducted to the excision of the plasmid resulting in a mutated *lacG* copy, as was confirmed by sequencing of PCR-amplified fragment spanning the mutated region.

### Disaccharide and monosaccharide analysis in culture supernatants

The cells from the *L. casei* cultures were eliminated by centrifugation and the supernatants were collected for sugar determination. The analyses were carried out by high-performance liquid chromatography (HPLC) with a Jasco PU2080Plus system. A Rezex RCM-Monosaccharide column (Phenomenex) (at 80 °C) was used and the samples elution was performed in isocratic mode using a flow rate of 0.6 ml min^−1^. The mobile phase was water and a refractive index detector (Jasco RI-2031 Plus) was used for carbohydrate detection. Peaks in the chromatograms were identified by comparing the retention times with those of the standards (LacNAc, lactose, glucose, galactose and GlcNAc).

### Expression and purification of His-tagged LacG

*lacG* coding region was amplified by PCR using genomic DNA of wild-type *L. casei* strain BL23 as template and the primers LacGBamHIFW (5′-TTTTGGATCCATGAGTAAACAGCTACCTCAAG) and LacGPstIRV (5′-TTTTCTGCAGTTAATCCGGAATGATGTGGG) with added *Bam*HI and *Pst*I restriction sites to the 5′- and 3′-ends (underlined). The obtained PCR product was digested with *Bam*HI and *Pst*I, cloned into pQE80 and the resulting plasmid pQElacG was used to transform *E. coli* BE50. DNA sequencing was carried out to confirm the correct construction. One clone, PE172 (pQElacG) was selected, grown in Luria-Bertani medium and induced with IPTG (1 mM) as described before^25^. The recombinant protein was purified and analyzed as described previously^24^.

### His-tagged LacG enzyme activity

100 μl reaction mixtures containing different *o*/*p*-nitrophenyl(NP)-sugars (Table 1) at 5 mM were performed in 96-well microtiter plates. The LacG activity was measured at 37 °C in 100 mM Tris-HCl buffer, pH 7.0, and the reaction started by adding 1 μg of enzyme. The amount of released *o*/*p*-nitrophenol was tested by tracking the absorbance change with time at 404 nm using a microplate reader (POLARstar Omega, BMG Labtech, Offenburg, Germany). The optimal pH and temperature reaction, and kinetic analysis were determined with *o*-NP-β-D-galactopyranoside-6-phosphate as previously described^24^.

LacG was tested for its capacity to hydrolyse different natural oligosaccharides (Table 1). 100 μl reactions containing substrate (4 mM) in 100 mM Tris-HCl buffer, pH7.0 were performed at 37 °C for 16 h. The reaction mixtures were analysed by HPLC as described above. Peaks in the chromatograms were identified by comparing the retention times with those of the standards (LNB, GNB, lacto-*N*-tetraose, lacto-*N*-neotetraose, 3′-*N*-acetilgalactosaminil-Gal, 3′-*N*-acetilglucosaminil-Man, 4′-galactofuranosil-GlcNAc, fucosyl-α1–3GlcNAc, LacNAc, lactose, lactulose, maltose, maltotriose, glucose, galactose, GlcNAc, mannose, GalNAc, L-fucose and fructose). All the oligo- and disaccharides were obtained from Carbosynth (Compton, Berkshire, UK), except lactose, lactulose, maltose and maltotriose that were obtained from Sigma-Aldrich (St. Louis, MO, USA).

### RNA isolation and Reverse Transcriptase quantitative PCR (RT-qPCR)

Total RNA was isolated from *L. casei* strain BL23 grown in sugar-free MRS supplemented with 4 mM of different sugars as previously described^24^. Cells were harvested at mid-exponential phase of growth (OD_550_ of 0.3 for cultures on glucose, galactose or GlcNAc and 0.5 for cultures on LacNAc, lactose, a mix of LacNAc plus glucose or lactose plus glucose). The isolated RNA was digested with DNaseI and retro-transcribed using the Maxima First strand cDNA Synthesis Kit (Fermentas)^24^. The resulted cDNA was subjected to quantitative PCR for the genes *lacT*, *lacE*, *lacG* and *lacF*. RT-qPCR was performed using the Lightcycler 2.0 system (Roche), LC Fast Start DNA Master SYBR green I (Roche) and primers pairs that produce amplicons ranging from 70 to 200 bp in size. RT-qPCR was performed for each cDNA sample in triplicate and using the primers pairs: qPCRlacTfor (5′-TTGTAAGGGGACGTGGCATC)/qPCRlacTrev (5′-TTGTCGGGAAGTCTCGTTCG) (*lacT*), qPCRlacEfor (5′-TTGGCCATGAACACGATGGA)/qPCRlacErev (5′-CCGAAAGTGCATGGCACAAA) (*lacE*), qPCRlacGfor (5′-AAGTCGAAGGAGCCACCAAG)/qPCRlacGrev (5′-GAACCGCCCCTGTTTATCCA) (*lacG*) and qPCRlacFfor (5′-GGTTTTGCACTTGTGGCGTA)/qPCRlacFrev (5′-CTGTTGGGCCTTCTCAACCA) (*lacF*). The reaction mixtures and cycling conditions were performed as previously described^24^. The *pyrG*, *lepA* and *IleS* genes were chosen as reference genes^68^. Cells growing in sugar-free MRS supplemented with glucose were used as reference condition. The relative expression based on the expression ratio between the target genes and reference genes was calculated using the software tool REST (relative expression software tool)^69^. The efficiency of all the primer pairs was between 1.9 and 2 (close to 100%). RT-qPCR reactions were performed in triplicate of two biological independent samples.

### Statistical analysis

Statistical analysis was performed using the Statgraphics Plus, ver. 2.1 (Statistical Graphics Corp., USA). One way analysis of variance (ANOVA) was used to assess the effects of the carbon source (galactose, GlcNAc, LacNAc, lactose, a mix of glucose and lactose, and a mix of glucose and LacNAc) on the expression levels of the *lac* genes. Student’s *t*-test was used to detect statistically significant differences between growth rate from *L. casei* wild-type BL23 strain versus each mutant BL393 (*lacAB*) and BL388 (*nagA*) strains. Statistical significance was accepted at *P* < 0.05.

### Data availability statement

All data generated or analyzed during this study are included in this published article.
